# Seeing other perspectives: evaluating the use of virtual and augmented reality to simulate visual impairments (OpenVisSim)

**DOI:** 10.1038/s41746-020-0242-6

**Published:** 2020-03-10

**Authors:** Pete R. Jones, Tamás Somoskeöy, Hugo Chow-Wing-Bom, David P. Crabb

**Affiliations:** 1 Division of Optometry and Visual Science, School of Health Science, Northampton Square, City, University of London, London, EC1V 0HB UK; 2 Institute of Ophthalmology, University College London (UCL), 11-43 Bath Street, London, EC1V 9EL UK; 3 NIHR Moorfields Biomedical Research Centre, London, EC1V 2PD UK

**Keywords:** Translational research, Public health, Vision disorders, Disability

## Abstract

Simulations of visual impairment are used to educate and inform the public. However, evidence regarding their accuracy remains lacking. Here we evaluated the effectiveness of modern digital technologies to simulate the everyday difficulties caused by glaucoma. 23 normally sighted adults performed two everyday tasks that glaucoma patients often report difficulties with: a visual search task in which participants attempted to locate a mobile phone in virtual domestic environments (virtual reality (VR)), and a visual mobility task in which participants navigated a physical, room-scale environment, while impairments were overlaid using augmented reality (AR). On some trials, a gaze-contingent simulated scotoma—generated using perimetric data from a real patient with advanced glaucoma—was presented in either the superior or inferior hemifield. The main outcome measure was task completion time. Eye and head movements were also tracked and used to assess individual differences in looking behaviors. The results showed that the simulated impairments substantially impaired performance in both the VR (visual search) and AR (visual mobility) tasks (both *P* < 0.001). Furthermore, and in line with previous patient data: impairments were greatest when the simulated Visual Field Loss (VFL) was inferior versus superior (*P* < 0.001), participants made more eye and head movements in the inferior VFL condition (*P* < 0.001), and participants rated the inferior VFL condition as more difficult (*P* < 0.001). Notably, the difference in performance between the inferior and superior conditions was almost as great as the difference between a superior VFL and no impairment at all (VR: 71%; AR: 70%). We conclude that modern digital simulators are able to replicate and objectively quantify some of the key everyday difficulties associated with visual impairments. Advantages, limitations, and possible applications of current technologies are discussed. Instructions are also given for how to freely obtain the software described (OpenVisSim).

## Introduction

Over 100 million people worldwide live with a chronic visual impairment (VI). The most common causes are glaucoma, age-related macular degeneration (AMD), and cataracts^1^. Unlike with long- or short-sightedness, the effects of VIs can be complex and highly heterogenous. Often, only a certain part of the visual-field is affected (e.g., predominantly peripheral vision in glaucoma, or predominantly central vision in AMD), and information in these regions is often not eliminated completely, but rather degraded in a variety of subtle ways: becoming blurry, faded, jumbled, or distorted^2^. Furthermore, symptoms may also vary markedly across individuals, eyes, and over time, with the sorts of visual symptoms a patient reports commensurate with the severity of damage.

Given the prevalence and complexity of VIs, simulations are often used to help communicate the day-to-day challenges that visually impaired individuals may experience. In medicine, simulations are used to educate the public, or to inform caregivers about a particular individual’s needs. In vision science, simulations are used to study the effects of VIs on tasks of daily living^3^. In health economics, an effective VI simulator has long been sought after as a way of informing health panels assessing the value-for-money of novel sight-loss treatments^4^. While in engineering, an effective simulator would aid in the design and assessment of more accessible products^5^ and built environments^6^.

**Fig. 1:**
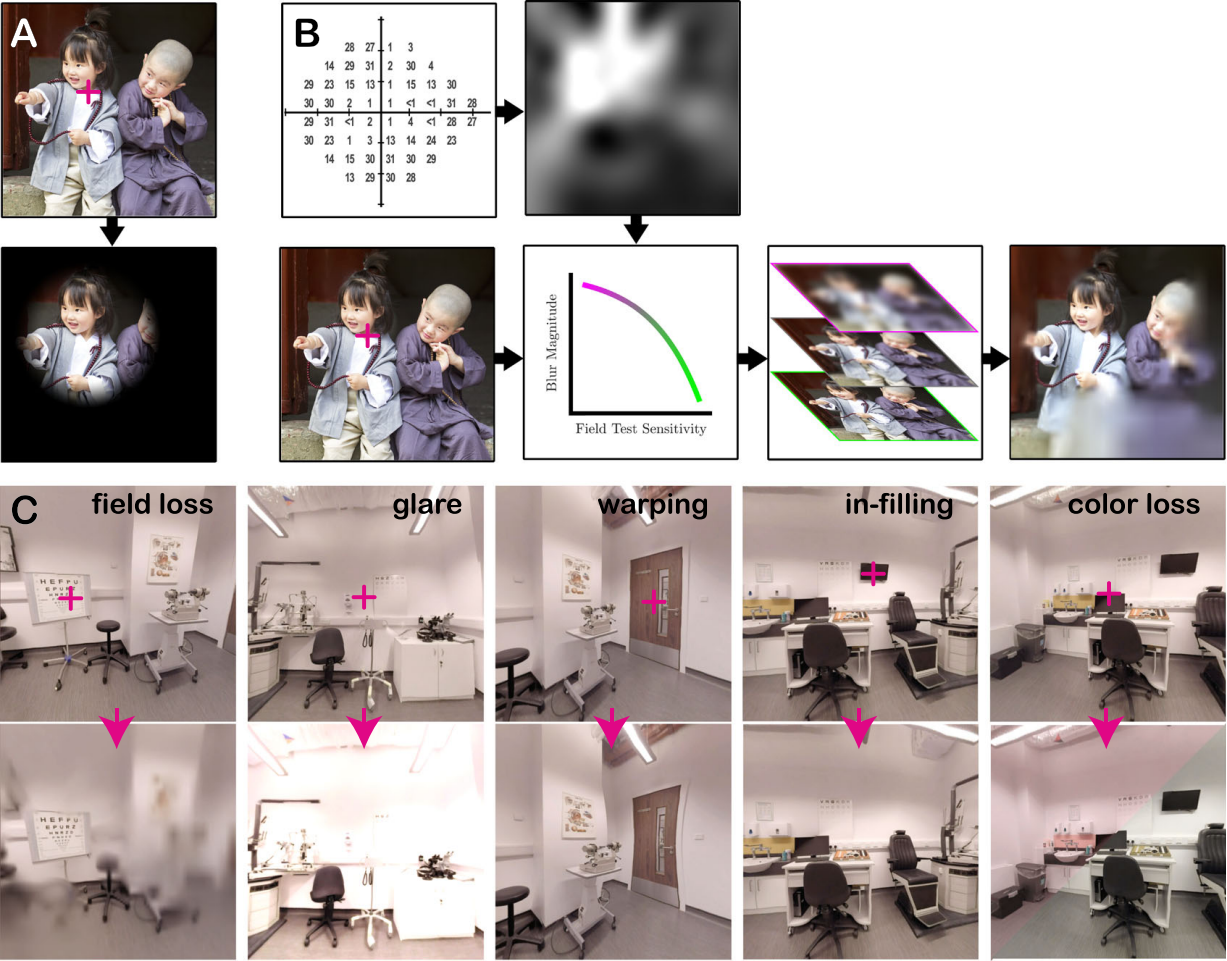
Simulations of sight loss. **a** Example of a conventional depiction of peripheral vision loss (e.g., glaucoma) using a crude method of occlusion. Such simulations are typically regarded as unrealistic by patients^2^; **b** Peripheral vision loss as simulated in OpenVisSim. Clinical visual field data from static automated perimetry is used to construct a gaze-contingent field map, which specifies the amount of blur to apply to each pixel. See Methods for details; **c** Examples of other potential image manipulations supported by OpenVisSim (not evaluated in the present paper). Additional visual disturbances, such as nystagmus, non-stationary warping, dynamic noise, and binocular misalignment (strabismus), are also supported, but cannot be easily illustrated using individual static images. For a video demonstrating many of these effects, see Supplementary Video 1.

Historically, VI simulators have consisted of either a static image or a pair of spectacles (‘SimSpecs), onto which a ‘black blob’ is superimposed to occlude a particular region of the visual field (see Fig. 1a). Such depictions are generally regarded as crude and unrealistic by patients, however^2,7^. They do not adequately reflect the range of symptoms that patients experience, and they allow the user to simply move their eyes to ‘look past’ the impairment. A more recent approach has been to apply a region of opacity to a contact lens^8^. Unlike SimSpecs, this allows the impairment to move with the eye (i.e., and so remains invariant on the retina, as with true VIs). However, the symptoms elicited by contact lenses also remain unrepresentative of real VIs: even a small region of opacity results in a diffuse shadow cast over the whole retina, rather than the localized degradations of form or spatial detail typically reported by patients^4^.

Recently, we described a more sophisticated simulator^9^ in which a Head Mounted Display (HMD) with integrated eye-tracking is used to perform gaze-contingent digital manipulations in virtual or augmented reality (VR/AR). The requisite hardware is widely commercially available, and, by using software that we have made freely available online (OpenVisSim), multiple different symptoms can be simulated simultaneously and in real-time (Fig. 1b; see Methods for how to obtain the software). Furthermore, each symptom can be parametrically manipulated, based on empirical eye-test data, and symptoms can be applied independently to each eye (Fig. 1c). While wearing the HMD, the user is free to walk around their environment, and to move their head and eyes. This means that users are able to perform the sorts of common, everyday actions that matter to patients, such as reading a newspaper or making a cup of tea.

Even the most sophisticated simulator will never be able to recreate exactly ‘what it is like’ to see through somebody else’s eyes. However, the question for the present work is whether these latest digital, gaze-contingent HMD simulations are sufficiently realistic to be of practical utility. We operationalized this question by asking whether OpenVisSim is capable of reproducing, in normally-sighted observers, the same basic patterns of difficulties that real glaucoma patients exhibit when faced with everyday tasks of daily living.

We focused on glaucoma for this initial assessment of the technology because it is one of the most common causes of irreversible sight loss worldwide^1^, but also because it is one of the most widely misunderstood^10–14^. It can be particularly hard for somebody with normal vision to imagine what a loss of peripheral vision is like, or why it is that an individual with healthy (‘20–20’) central vision is nonetheless more likely to fall^15^, to be involved in a car accident^16^, or to live a more sedentary and restricted life^17^. A further advantage is that glaucoma has been widely studied, and the difficulties faced by glaucoma patients are well characterized. In brief, individuals with glaucoma often report particular difficulty locating objects in cluttered visual scenes^18^, and also exhibit reduced mobility^17^ and increased risk of falls^15^. Furthermore, these difficulties tend to be most pronounced when the loss occurs in the inferior visual field, compared to when the loss of vision occurs above the midline^19,20^. It has also been shown that individuals with glaucoma tend to make more eye- and head- movements, to compensate for their restricted field of view^21–23^.

**Fig. 2:**
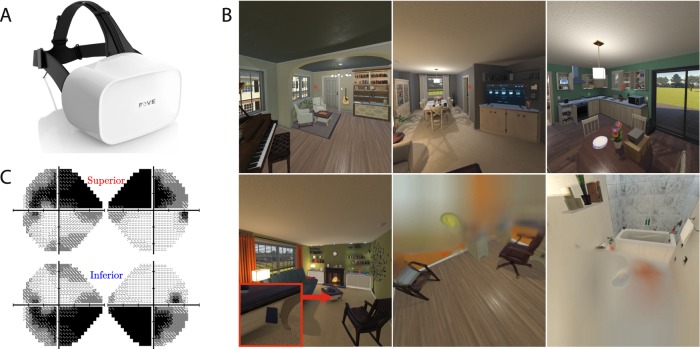
VR search task methods. **a** Hardware: The Fove0 VR headset with integrated eye tracking, which was worn throughout. **b** Task: Example screenshots from the VR simulator, while performing the Object Search task. Virtual rooms were configured into a complete ‘suburban style’ house. Depending on the player location, it was often possible to see into other rooms, connecting hallways, etc. For added realism the outdoor scenes were also rendered and could be seen from those rooms with external windows (see top-right panel). On each trial, the target phone (highlighted in the bottom-left panel) was placed at a random location. Examples of the superior and inferior VFL (right-eye only) are depicted in the bottom-middle and bottom-right panels, respectively (NB: the red crosshair indicates current their point of fixation and was not visible to participants). **c** The empirical perimetric data used to generate the simulated VFL. The superior field-loss measurements were made using Static Automated Perimetry, using a single observer with an established diagnosis of glaucoma (see Methods). The inferior loss is the exact mirror inverse of the superior. See Supplementary Video 2 for an example trial.

**Fig. 3:**
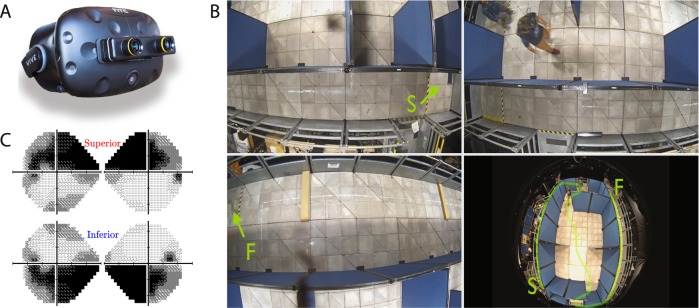
AR Visual Mobility task methods. **a** Hardware: The modified HTC Vive headset, with ZEDmini stereoscopic cameras and integrated Tobii eye-tracking. **b** Task: Screenshots from four simultaneous camera feeds, recorded during a typical trial of the Visual Mobility task. The participant’s task was to trace an approximately ‘s’ shaped route (green line) from the start point [S] to the finish point [F]. The configuration of the blue partitions, and the exact location of the two foam obstacles (shown in bottom left panel), was varied randomly between trials. An experimenter followed the observer at a safe-distance in case of any trips or accidents. See Bainbridge et al.^59^ for further details. **c** The simulated VFL conditions were the same as in the main experiment (Fig. 2). See Supplementary Video 3 for an example trial.

To examine whether these phenomena could be elicited by a simulated impairment, we asked normally-sighted adults to perform two everyday tasks. One was an Object Search task, in which they attempted to locate a smartphone in a virtual house (Fig. 2). The other was a Visual Mobility task, in which the participant attempted to navigate a real physical environment, using AR (Fig. 3). Both were performed with and without simulated visual field loss (VFL). The simulated vision loss was based on perimetric data from a real patient with glaucoma, and was located in either the inferior or superior hemifield. If the simulator is capable of functionally approximating the true glaucoma patients experience, then both forms of VFL should elevate search times, but the effect should be greatest when the VFL was inferior. To examine whether the VI also caused changes in looking behaviors, eye- and head- movements were also recorded using the near-infrared and gyroscopic sensors contained within the HMD.

## Results

### Performance: VR visual search

**Fig. 4:**
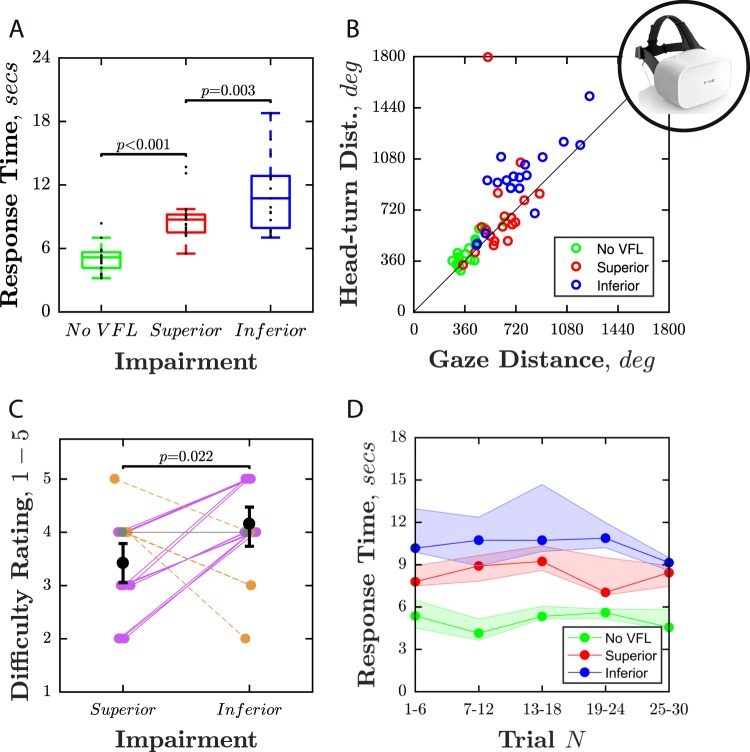
Performance on VR search task. **a** Response time as a function of impairment type (box-and-whisker plots, showing median, interquartile range, and range). Black dots indicate individual participants. *P* values relate to Wilcoxon signed-rank tests (i.e., non-parametric repeated measures *t*-tests; see body text for statistics). **b** Scatter plot showing the cumulative distance traveled by each participant’s head and eyes in a single trial, averaged across all trials. Note the separation between each of the three conditions. **c** Self-reported difficulty ratings. Lines indicate individual participants, with those who rated the Inferior condition as more difficult highlighted by purple solid line. Black bars indicate group-means and 95% confidence intervals (CI_95%_). **d** Group-median response times, ±CI_95%_. These data are the same as those in panel A, but are here broken down into individual six-trial blocks to assess the consistency of the effect, and possible learning or fatigue.

Compared to No VFL (Visual Field Loss), median search times were 74% slower in the Superior VFL condition (+3.7 secs; Wilcoxon signed-rank test: *z* = −3.82, *P* < 0.001), and 125% slower in the Inferior VFL condition ( + 6.3 secs; *z* = −3.82, *P* < 0.001). Search times were significantly slower for Inferior VFL than Superior VFL (*z* = −3.02, *P* = 0.003; Fig. 4a), and the difference ( + 2.6 secs) was 70% as great as the difference between the Superior condition and No VFL ( + 3.7 secs). Participants also made more head movements (*z* = −2.37, *P* = 0.017) and more eye movements in the Inferior VFL condition (*z* = −3.09, *P* = 0.002) versus the Superior condition (Fig. 4b), and also rated the Inferior VFL condition as more difficult (*z* = −2.29, *P* = 0.022; Fig. 4c). The differences in search time observed between the three conditions occurred consistently throughout the testing session, with no compelling evidence of learning or fatigue (Fig. 4d).

### Individual variability

There was considerable individual variability in how well participants coped with the presence of simulated VFL. Thus, while all participants were slower in the two VFL loss conditions than in the No VFL condition, mean response times increased by between 7 and 134% for the Superior impairment (mean [SD]: 80% [41%]), and by 52–220% for the Inferior impairment (mean [SD]: 125% [46%]).

**Fig. 5:**
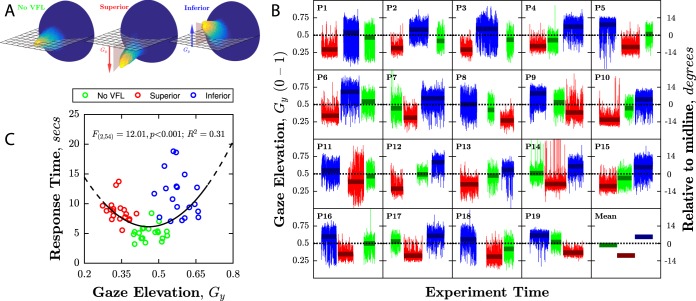
Looking behaviors during VR search task. **a** Eyeball orientation in each condition. Each plot is a 3D histogram showing the estimated gaze vector, as measured every 8.3 msec throughout the experiment (~500,000 data points per plot, data averaged across eyes, data from all 19 participants included). These histograms show that participants tended to look downwards in the Superior VFL condition, and upwards in the Inferior VFL condition. **b** Gaze elevation (in normalized screen units, 0–1) as a function of time for each participant, and the group-mean value across all participants (bottom-right). The 0.5 point indicates the midline of the head-mounted display screen (central fixation). A value of 0.75 indicates fixation around the upper quarter of the screen (+14° above the midline). **c** Response time as a function of gaze-elevation. Circles represent individual participants (one data-point per participant, per condition). The black curve indicates the best-fitting quadratic model (weighted sum of squares, fitted using bisquare weights).

These individual differences in performance were associated with systematic differences in gaze behavior. Thus, as shown in Fig. 5, participants tended to fixate around the midline in the No VFL condition, but fixated consistently higher in the Inferior VFL condition (*z* = 3.82, *P* < 0.001), and consistently lower in the Superior VFL condition (*z* = −3.70, *P* < 0.001; Fig. 5a). This pattern was observed in all 19 individuals (Fig. 5b). However, those observers who modified their gaze least, and continued to fixate more centrally in all conditions, performed faster in the impairment conditions (Quadratic regression; *F*_(2,54)_ = 12.01, *P* < 0.001, *R*^2^ = 0.31; Fig. 5c). This suggests that observers who utilized more adaptive viewing strategies were better-able to cope with the exact same vision loss.

### Performance: AR visual mobility

**Fig. 6:**
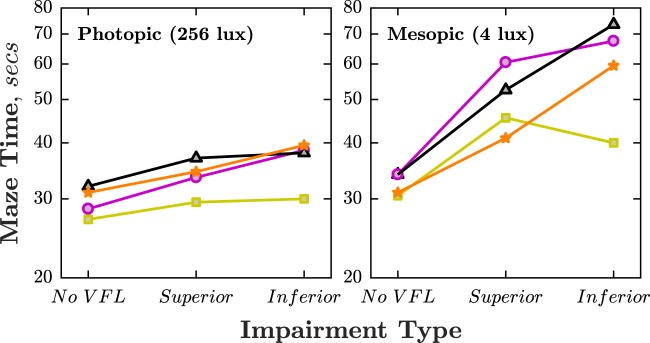
Performance on AR visual. Panels show maze completions times under **a** Photopic and **b** Mesopic lighting conditions. Each line indicates a single participant (same individuals in both conditions).

The pattern of results in the AR mobility task was the same as in the main VR visual search task. Participants were slower to complete the maze when vision was impaired, but were particularly slow when the impairment was Inferior (Fig. 6). Compared to the No VFL condition, participants were 13%/52% (photopic/mesopic) slower in the Superior VFL condition, and 23%/95% (photopic/mesopic) slower in the Inferior VFL condition. Under photopic lighting, the difference between the Superior and Inferior conditions (+3.0 s) was 71% as great as the difference between the Superior VFL condition and No VFL (+4.3 s). Under mesopic lighting, the difference between the Superior VFL and Inferior VFL conditions (+13.0 s) was 76% as great as the difference between the Superior VFL condition and No VFL (+16.8 s). As in the main VR experiment, however, there was substantial individual variability in performance (see Fig. 6).

## Discussion

The present study examined whether gaze-contingent simulations of visual impairment (OpenVisSim), presented using a head mounted display (HMD), are capable of eliciting in normally-sighted observers the sorts of everyday difficulties experienced by real glaucoma patients. The results were encouraging. As with real glaucoma patients, participants were slower to perform everyday visual-search (VR) and mobility (AR) tasks when experiencing simulated VFL, and as with real patients these difficulties were exacerbated when the VFL was inferior. Furthermore, as with real patients^21–23^, participants made more head- and eye-movements when experiencing VFL, to compensate for their restricted field of view. Taken together, these results suggest that mixed reality (AR/VR) technologies have interesting potential as a means of simulating the functional effects of VI in normally-sighted individuals. This could have wide ranging practical applications, as detailed below.

Anecdotally, it was also noticeable that, in addition to the objective changes in performance, many participants reported feeling anxious when the impairment was active. This was particularly the case when participants were ascending/descending the stairs that led to the AR mobility platform. Interestingly, “climbing stairs” is also a regular source of anxiety for many people with severe vision loss^24^, and these ‘psychological’ aspects of visual impairment can also have a substantial impact on wellbeing. For example, elevated levels of depression and physical inactivity are common among the visually impaired^25^, and in extreme cases can lead to individuals being afraid to leave their own homes^17^. The fact that the simulator was able to elicit these psychological components was unanticipated but encouraging, and could be explored more systematically in future.

That inferior deficits are more detrimental for performing some visually guided actions is consistent with previous self-reports from real glaucoma patients^19,26,27^. Unlike with self-report data, however, the use of VR/AR furthered allowed us to quantify effect sizes, and explore individual differences. With regard to effect size, the presence of a severe superior VFL caused average response times to increase by around 50% (VR Search: 74%; AR Mobility: 54%), versus no impairment. Shifting the scotoma from a superior to inferior location caused response times to increase by over half as much again (VR Search: +70%; AR Mobility: +71%). This indicates that the retinotopic location of an impairment can be almost as important as the difference between advanced VFL and having no impairment at all. Regarding individual differences, there was considerable variability in levels of impairment exhibited (Fig. 3c). For example, in the VR search task, response times increased by 52–220% across participants in the Inferior VFL condition, even though the simulated loss was identical for all. This supports the intuition that some individuals are better at coping with same level of sight loss, and shows that this reflects not just differences in psychology^28,29^ or lifestyle^30^, but also represents genuine differences in capability. The best performing individuals tended to be those who continued to maintain relatively normal gaze behaviors even when experiencing VFL, and it is tempting to speculate whether the present technology could be adapted to ‘teach’ such adaptive strategies in future.

It would be wrong, on the basis of the present results, to conclude that inferior VFL is always worse, and it may be that for other tasks a superior scotoma is more debilitating. For example, several studies have reported that superior VFL is more detrimental for particular tasks such as driving^26,31–33^. One of the benefits of the present approach is that it can be easily adapted to explore a range of everyday scenarios. For example, tasks could be implemented that map onto each of the categories of behavior defined in the NEI-VFQ-25^34^ or GQL-15^35^ (e.g., near-vision, distance-vision, driving, etc.).

The present technology could also be used to simulate other VFL profiles, or to explore the needs of a particular patient. For example, the VF data in the present study was from a single individual with overlapping (binocular) VFL in each eye. However, we are also using the same paradigm to explore, for example, how a purely unilateral deficit effects everyday vision-related tasks^36^.

The size and location of the simulated visual field loss was consistent with clinical data, and the use of blur to degrade the image was consistent with the qualitative reports of patients^2^. However, the simulations described in the present work were only intended as a first-approximation of glaucomatous sight loss. The simulator could be improved in future by incorporating additional features, such as spatial distortions and ‘filling-in’ effects: aspects of glaucoma that are sometimes reported by patients, but which we currently lack the means to quantify robustly. An ideal simulator would also take into account the fact that, for many patients, the extent/quality of their vision loss varies, depend on their own physiological state or their current viewing conditions (e.g., level of ambient illumination). Thus, it has been observed that some glaucoma patients exhibit a greater loss of vision under home-lighting, than when assessed in a well-lit eye clinic^37,38^.

Some additional image manipulations are already supported by the simulator (see Fig. 1c), but were not employed in the present work, in part due to a lack of appropriate clinical data with which to constrain them. Some other visual phenomena are difficult to simulate using current hardware. This is most notably the case with changes in contrast sensitivity—a common complaint of many VI patients^35^, but one that is computationally challenging to depict accurately, as it requires the simulator to effectively model an ascending hierarchy of increasing receptive fields, as exist within the visual system^39^. It would be helpful in future to develop algorithmic approximations of contrast sensitivity loss, or specialized hardware capable of quantitatively simulating such deficits in near-real-time within VR/AR. In order to facilitate further development, we have made the complete codebase for our simulator freely available online (https://github.com/petejonze/OpenVisSim). Technically minded readers are encouraged to adapt or modify the code, and can contribute changes to the online repository. Furthermore, the code is written in a popular games engine (Unity3D), which means that it is compatible with all modern hardware (including smartphones), and can be easily integrated into many existing software packages (e.g., driving simulators).

This study was intended only as an initial assessment of feasibility: designed to explore the raw potential of using a head mounted display (HMD) to deliver digital, gaze-contingent simulations of visual impairment. A much larger sample, and a more standardized protocol would be required to formally assess the accuracy and/or utility of this new technology. It would also be desirable to explore the usability and acceptability of the technology in older adults, since the prevalence of eye-disease increases greatly with age^40^.

It should also be noted that participants in the present study experienced an extremely acute onset of vision loss. As a result, they most likely experienced a ‘positive’ scotoma (a salient obstruction/alteration in the visual field). This stands in contrast to many real forms of VI, where vision loss occurs gradually over many years^41^, and where, due to a gradual process of adaptation, patients often report a ‘negative’ scotoma (an imperceptible absence of information). There was no indication that task performance or looking behaviors changed over the brief period of the experiment (see Figs 4d and 5b). However, in future it would be instructive to explore whether performance, and/or people’s perceptions of VFL, changed following a longer and progressive period of simulated sight loss. In this respect, the possibilities afforded by AR are particularly exciting, as such devices could foreseeably be worn for days or weeks. It would be particularly interesting to examine, for example, whether the human visual system is able to recover from chronically altered signals, in a similar way as has been reported previously in audition^42^, and in vision using prisms^43^. Likewise, AR could be used to explore whether prolonged simulated sight-loss leads to subtle changes in the ‘microstructure’ of eye-movements (e.g., rate of corrective saccades), as have been shown previously to occur following glaucoma^21,44^.

The VI simulator described in the present work combines: (i) real-time, gaze-contingent image manipulations; (ii) clinical data; and (iii) stereoscopic presentation via a HMD. Considered in isolation, these constituent elements are not unique. The core algorithm for simulating visual field loss in real-time was first proposed by Geisler and Perry^45^. The idea of using data from ophthalmic eye-tests to generate clinically relevant impairments has been proposed most convincingly by Thompson et al (2017)^39^. And many people have developed VR/AR sight-loss simulators^46–49^ of varying technical sophistication. To our knowledge, however, this is the first system to integrate these elements together into a single, functioning system, and it is the first to be shown capable of eliciting plausible impairments on the sorts of typical, ‘real world’ tasks of daily living that patients really value (i.e., rather than purely perceptual changes in the user’s ability to recognize words or letters^39,46^). We anticipate that the present findings would generalize to other similar system, should any be developed in future, and that the realism of such systems will only increase as the hardware and algorithms continue to develop.

As highlighted in the Introduction, the ability to simulate VI has many potential applications across science, engineering, and medicine. In clinical science, an effective simulator could be used to improve public understanding of visual impairments^50^: current low levels^10–14^ of which are thought to be a key driver behind current high rates of late diagnosis across many diseases^51^. A synthetic testbed could provide novel insights that would be impractical to obtain from real patients. For example, while it is already well-established that patients with inferior VFL are more likely to report difficulties with everyday tasks than their peers with superior VFL^19^, in this study we were able to quantify the size of this effect, and in so doing, show that the difference between a superior and inferior scotoma can be almost as great as the difference between a severe superior scotoma and no impairment at all.

With regards to engineering, sight-loss simulators could be instrumental in ensuring that products^5^ and built environments^6^ are accessible to individuals with reduced vision. This is increasingly becoming a legal requirement in many countries. However, regulatory requirements have focused traditionally on physical disabilities (e.g., ensuring step-free access, and doorways wide enough to accommodate wheelchairs). This is understandable, as the challenges arising from sensory impairments are complex and hard to codify. What is particularly exciting about the VR/AR approach proposed in the present work is that it makes visual accessibility a straightforward, empirical question: architects and engineers can observe directly what works for users with reduced vision, and what does not. Often, the necessary changes may be relatively small, but such changes can nevertheless be highly meaningful for people with reduced vision. And these may include purely perceptual factors (e.g., improving lighting, and increasing the contrast of signage), but also higher levels considerations, such as making an environment more predictable. For example, in the VR search task it was observed during piloting that effects of VFL were further exacerbated if the phone was allowed to appear in unexpected locations (e.g., inside toilet bowls, or on the ceiling), or if contextual information were removed altogether by replacing the target and environment with randomly textured noise.

In health economics, an effective sight-loss simulator could also be helpful when determining the financial value of a given treatment. Thus, one difficulty often faced is how to quantify the relative cost/benefit of qualitatively distinct interventions—from chemotherapy for lung cancer, to surgery for glaucoma, or intravitreal injections for macular degeneration. These health economic judgments often center on questions such as: how does the benefit of preserving peripheral vision compare to an additional eighteen months of life expectancy? The official position of health-care standards authorities, such as the National Institute for Health and Care Excellence (NICE) in the UK, is that such questions should be answered by ordinary, unbiased members of the general public^52^. The difficulty, however, is that the public generally has poor comprehension of what life with a visual impairment is really like^10–14^, leading to decisions that may fail to maximize societal health benefits^53^. The failure in the past to simulate vision loss using spectacles or contact lenses has led some economists to call for precisely the sort of gaze-contingent, real-time, digital simulator that we describe here^4^.

More generally, the present work highlights the possibility of using VR/AR as a new way of evaluating the impact of sight loss on the sorts of everyday tasks that patients really value. The present results are promising in that we were able to robustly evidence changes in both performance and looking behaviors (i.e., eye- and head-movements), and did so in manner that was safe, convenient, replicable, and precisely controlled. In this sense, VR/AR—as has been recently noted recently by others^54^—can be seen as the logical extension of traditional ‘performance based’ assessments^55^ such as the Assessment of Visual Disability Related to Vision (ADREV)^56^. However, direct application of VR/AR to patients will only be appropriate once headsets are developed that are more comfortable and lightweight, and once they routinely support effective refractive correction^57,58^. Once these technical challenges are solved, however, the potential rewards are considerable: opening up a powerful new way to objectively assess the real-world impact of sight loss.

## Methods

### Participants

Participants were 23 normally sighted adults aged 18–40 (Median = 26.5) years. Nineteen performed the main VR search task, while four performed a secondary AR Visual Mobility task. In all cases, normal vision was confirmed by letter acuity (equal to or better than 6/12), standard automated perimetry (“within normal limits” on a Glaucoma Hemifield Test using a Humphrey Field Analyzer 24-II SITA Fast program; Carl Zeiss Meditec, CA, USA), and the Wirt Stereo Fly Test (≤80 s). No assessment of far-peripheral vision was performed, although this could be done in future using wide-field retinal-imaging or kinetic perimetry. Participants received £15 compensation, and were recruited by adverts placed around City, University of London.

### Ethics

All participants provided informed written consent. All experiments were conducted in accordance with the declaration of Helsinki, and followed ethical approval from City, University of London’s School of Health Sciences (#Opt/PR/16–17/58).

### Simulated VFL

To simulate glaucomatous visual field loss (VFL) we created a gaze-contingent region of variable blur using OpenVisSim (see Fig. 1b and Supplementary Video 2 for examples; see Supplementary Methods and Jones and Ometto^9^ for technical details). Although only intended as a first approximation, blur is the most consistently reported symptom of glaucoma patients^2^, and is prima facie consistent with more sparse sampling of the scene due to ganglion cells loss and/or dysfunction. The magnitude and shape of the blur field was determined by clinical data from a 68-year-old with an established diagnosis of glaucoma (Fig. 2c). Note that the simulator also supports a variety of other degradation effects (see Fig. 1c), including as spatial distortions (metamorphopsia), ‘filling-in’ effects, and color vision deficits. These additional effects were not employed/evaluated in the present work, however, as they are less commonly reported in glaucoma, and because we generally lack appropriate quantitative, clinical data with which to constrain them. Crucially, the location of the VFL on the screen was updated in near-real-time based on the participants current point-of-gaze, and so remained static on the observer’s retina, irrespective of eye- or head-movements. The Inferior VFL was identical in every respect to the superior VFL, except flipped up-down along the horizontal meridian. The complete source code for generating the simulated impairments can be found at: https://github.com/petejonze/OpenVisSim, and is free for non-commercial use.

### Primary task: visual search in virtual reality

To assess the impact of the simulated VFL on people’s ability to locate an object in a cluttered visual scene, 19 participants were asked to find a mobile phone located around a virtual house (Fig. 2; Supplementary Video 2). Fifteen domestic environments (rooms) were created (see Fig. 2b), and these were rendered using Unity3D (Unity Technologies ApS, San Francisco, CA, United States). Environments were viewed stereoscopically using a virtual reality headset with integrated eye-tracking (FOVE Inc., San Mateo, CA, United States). On each trial, one of the fifteen virtual rooms was randomly selected, and the location of the phone, the location of the participant, and the starting orientation of their head was randomized (NB: values constrained so that the participant was never directly facing the target at trial onset). In actuality, participants were seated on a rotating office chair throughout, but they could turn their body freely (360°) and were free to move their head and eyes to look around the virtual environment. Participants pressed a button to indicate when they had found the phone, and responses were verified as correct only if the participant’s gaze fell within 45**°** of the target (NB: This 45° criterion was intended only to filter out occasional ‘finger press’ errors, and participants were monitored throughout to ensure they were performing the task correctly). The primary outcome measure was response time. Eye and head movements were also tracked using the headsets near-infrared and gyroscopic sensors.

Participants completed three blocks of 30 trials (90 trials total; ~45 min, including breaks). Each block tested a single condition (No VFL, Superior VFL, Inferior VFL). The order of conditions was randomized between participants. Before the first block, participants were shown the target phone, and completed 10 practice trials (No VFL).

### Secondary task: visual mobility using augmented reality

Glaucoma is also associated with reduced mobility and increased risk of falls. To confirm that any effects generalize beyond the main virtual-reality search task, we also asked four new participants to perform a real-world mobility task, in which they navigated the maze shown in Fig. 3 (a physical environment constructed previously to evaluate the efficacy of gene therapies for inherited eye-disease^59^). The simulated VFL conditions were identical to those in the primary search task: the only difference being that the raw input to each eye was not computer generated, but instead provided by a pair of forward-facing stereoscopic cameras (Augmented Reality). Again, the hypothesis was that performance would be worst (slowest) when the VFL was inferior.

Note that fewer participants (*N* = 4) took part in this task than in the main VR experiment (*N* = 19). This reflects the much greater costs—both in terms of time and money—associated with ‘real-world’ testing, as well as the fact that the results were only intended to confirm and extend the results from the primary experiment. Participants completed the task twice under photopic conditions equivalent to ordinary office lighting (256 lux), and twice under mesopic conditions (4 lux; UK nighttime pedestrian lighting standard).

### Reporting summary

Further information on research design is available in the Nature Research Reporting Summary linked to this article.

## Supplementary information

Supplementary Information

Reporting Summary

Supplementary Video 1

Supplementary Video 2

Supplementary Video 3

## Data Availability

All data required to evaluate the conclusions in the paper are present in the paper and/or the Supplementary Materials. Additional data available upon request.

## Code availability

The complete source code for generating the simulated impairments can be found at: https://github.com/petejonze/OpenVisSim, and is free for non-commercial use. The code and materials for running the VR experiment requires additional, third-party licenses, and is available on request.
