# Supplementary material for: Seeing other perspectives: evaluating the use of virtual and augmented reality to simulate visual impairments (OpenVisSim)
Source: NPJ Digit Med. 2020 Mar 10;3:32. doi: 10.1038/s41746-020-0242-6 (PMC7064490; doi:10.1038/s41746-020-0242-6)
Supplement: Supplementary file 1 — Supplementary Information [file 41746_2020_242_MOESM1_ESM.pdf]

Supplementary Information

Jones, Somoskeöy, Chow-Wing-Bom, & Crabb

S1. Supplementary Videos

- Supplementary Video 1: *Example outputs from the OpenVisSim visual impairment simulator*
- Supplementary Video 2: *Example trial from the virtual reality (VR) visual search task*
- Supplementary Video 3: *Example trial from the augmented reality (AR) mobility task*

S2. Supplementary Methods

S2.1. Software

Simulations of visual field loss (VFL) were implemented independently in each eye, and consisted of a gaze-contingent region of variable blur. For the Superior VFL condition, the shape of the VFL in each eye was derived as follows from monocular static threshold perimetry data from a single individual with an established diagnosis of glaucoma.

First, individual Differential Light Sensitivity (DLS) values were interpolated and extrapolated from a 24-2 grid using a hierarchical/multilayer radial basis function model (RBL-ML), fitted using the ALGLIB toolbox (ALGLIB Project, Nizhny Novgorod, Russian Federation). Note that this test grid only measures DLS values across the central 48 degrees of the visual field, whereas most VR headsets have a Field Of View [FOV] of $\sim 100°$ or more. Values outside the tested range were extrapolated by linear interpolation. This is a crude approach, however, and it would be desirable in future to explore more robust methods of visual field extrapolation.

Next, as illustrated in Figure 1B of the main manuscript, the interpolated/extrapolated DLS values were used to blur the image on the screen using the multiresolution pyramid approach proposed previously by Geisler and Perry (2002)[1]. In brief, before every screen refresh, the source image was converted into a hierarchy of increasingly blurred variants, by repeatedly smoothing the image and down-sampling by a factor of two. The magnitude of blur could be controlled pixel-by-pixel, by sampling from different level of this hierarchy: interpolating between adjacent levels as required. Unlike Geisler and Perry, the pyramid was not computed explicitly, but was generated implicitly as part of the default OpenGL pipeline. This further improved the efficiency of the algorithm, and meant that the computational overhead was minimal (crucial for gaze-contingent viewing, or for AR applications).

The magnitude of blur at each pixel location was proportional to the severity of visual field loss in retinotopic (gaze-contingent) coordinates (interpolating between measurement locations, as required). The level of blur was sufficient to cause a substantial visible scotoma in regions of severe VFL loss (see video). Note that blur primarily reduces acuity, whereas perimeters measure contrast sensitivity. In practice, however, the two are correlated[2] (albeit imperfectly), and as a first-approximation the amount of blur was scaled to produce the approximate degree of sensitivity loss predicted by Asaoka (2013)[2].

For further technical details on this, and other features of the OpenVisSim simulator not evaluated in the present work (e.g., color vision loss, spatial distortions, in-painting effects), see Jones & Ometto (2018)[3].

S2.2. Hardware

In Experiment 1 (VR), graphics were displayed on a FOVE0 Eye-Tracking VR headset (FOVE Inc., San Mateo, CA, United States). This contains a 2560 X 1440 WQHD OLED panel (1280 x

1440 pixels per eye), with a refresh rate of 70Hz and a binocular field of view of approximately 100 degrees. In Experiment 2 (AR), graphics were displayed on a modified HTC Vive headset (HTC Vive; HTC Corporation Limited, New Taipei, Taiwan, with forward-facing ZEDmini stereoscopic cameras (Zed Mini Stereo Camera, Stereolabs Inc., San Francisco, USA), and integrated Tobii eye-tracking (Tobii Pro HTC Vive Integration; Tobii AB, Stockholm, Sweden).

Both headsets contained near-infrared eye-trackers (1 per eye) allowing gaze to be independently monitored in each eye, with a single-frame precision of approximately 1 deg, and a refresh rate of 120 Hz. Both headsets also contained inertial sensors (gyroscope, accelerometer) for monitoring head-pose. There was no crosstalk between the two eyes, as stimuli — and simulated impairments — were presented dichoptically.

In Experiment 1, the software was controlled by a HP OMEN laptop (Hewlett-Packard Company, Palo Alto, CA, United States) containing a NVIDIA GTX 1050Ti graphics card (NVIDIA Corp, Santa Clara, CA, United States). For portability, this was exchanged for a backpack computer in Experiment 2 (MSI VR ONE 6RD-009 Backpack PC; Micro-Star International Co. Ltd, New Taipei, Taiwan), containing an NVIDIA GeForce GTX 1060 graphics card.

Due to the highly efficient nature of the blurring algorithm (see above), display lag was primarily a function of the hardware. Thus, the transmission time from the eye-tracking hardware was on the order of 20 msec. If we further factor in the refresh rate of the screen (70 Hz) and 3D rendering time, total expected lag was approximately 30-40 msec (well below the duration of a typical saccade). It is unknown whether this lag affected the present results, though subjectively the simulations felt relatively smooth and responsive.

Before the start of the experiment, the eye-tracker was calibrated using the manufacturer-supplied procedure. This was then validated, both by the software's own internal algorithms, and by an informal process of inspection in which the experimenter manually manipulated the location of a target (a red dot), and observed the participant's estimated gaze location. If the headset reported poor calibration, or if the experimenter was not completely satisfied with its accuracy, the calibration was re-run. This happened on 1% of occasions, generally if the participant physically adjusted the position/straps of the head-mounted display during calibration. During testing, estimated gaze was also visualized on a separate screen, overlaid onto the visual scene. The experimenter monitored this screen for any unusual gaze behavior, and could manually trigger a recalibration. In practice, however, no interventions were required.

SUPPLEMENTAL REFERENCES

[1] Geisler, W. S. & Perry, J. S. Real-time simulation of arbitrary visual fields. In *Proceedings of the 2002 symposium on Eye tracking research & applications*, 83–87 (ACM, 2002).

[2] Asaoka, R. The relationship between visual acuity and central visual field sensitivity in advanced glaucoma. *The British Journal of Ophthalmology* **97**, 1355–1356 (2013).

[3] Jones, P. R. & Ometto, G. Degraded reality: Using vr/ar to simulate visual impairments. In *2018 IEEE Workshop on Augmented and Virtual Realities for Good (VAR4Good)*, 1–4 (2018).
